# Dual atrio ventricular node in heterotaxy syndrome

**DOI:** 10.1002/joa3.12525

**Published:** 2021-04-08

**Authors:** Debasis Acharya, Jogendra Singh, Debasish Das, Rama Chandra Barik, Dibya Ranjan Behera, Dibya S. Mahanta

**Affiliations:** ^1^ Department of Cardiology All India Institute of Medical Sciences Bhubaneswar India

**Keywords:** heterotaxy syndrome, reentrant supraventricular tachycardia

## Abstract

Our interesting electrocardiogram has two qRS morphology without features of preexcitation suggesting two atrio ventricular node conduction system. All cardiologists should be aware of this feature in heterotaxy syndrome as reentrant supraventricular tachycardia may develop in these patients.

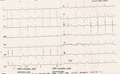

Twelve‐year‐old male diagnosed as heterotaxy syndrome with complete atrioventricular canal defect planned for intracardiac repair presented with the following electrocardiogram (ECG; Figure 1).

**FIGURE 1:**
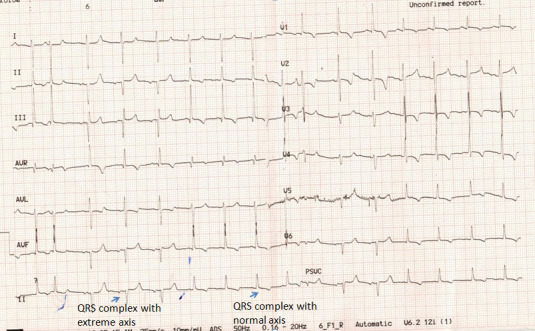
Electrocardiogram showing sinus rhythm with two different types of qRS morphology marked by arrow. First marked qRS has extreme axis and second marked has normal axis

The ECG has sinus rhythm with normal PR interval of 110 m sec and two types of qRS morphology with different axis. One group of qRS has normal axis and other has extreme axis (Marked arrow). Both types of qRS are narrow and hence non preexcited. There are no features of any hemiblock. So most probably ventricle is activated in two different conduction system which are oriented in different direction. There are reports of presence of dual atrio ventricular (AV) node in some complex congenital heart disease most commonly with heterotaxy syndrome with right atrial isomerism and some cases of discordant atrioventricular connection.

One study using 3D electroanatomic mapping system in patients of heterotaxy syndrome with atrioventricular septal defect has showed the presence of two AV nodes one at the superior and other at inferior aspects of the common AV junction connected by a sling of conduction tissue along the ventricular border of the AV septal defect. They have shown the presence of two separate discrete His potential decremental and adenosine sensitive conduction and inducible AV reciprocating tachycardia involving both AV nodes.^1^ Reentrant supraventricular tachycardia can develop involving one AV node as anterograde and other as retrograde limb and can be treated by ablating one of the node.^2^

## CONFLICT OF INTEREST

The authors declare no conflict of interests for this article.
